# Laminopathies

**DOI:** 10.1080/19491034.2018.1515606

**Published:** 2018-12-17

**Authors:** Giovanna Lattanzi, Lorenzo Maggi, David Araujo-Vilar

**Affiliations:** CNR Institute of Molecular Genetics, Unit of Bologna, Bologna, Italy; Rizzoli Orthopedic Institute, Bologna, Italy; Neurology IV - Neuroimmunology and Neuromuscular Diseases Unit, Fondazione IRCCS Istituto Neurologico “Carlo Besta”, Milan, Italy; UETeM-Molecular Pathology Group. Department of Medicine, IDIS-CIMUS, University of Santiago de Compostela, Spain

Laminopathies are tissue-specific or systemic disorders caused by mutations in *LMNA* gene encoding lamin A/C (primary laminopathies) or in genes encoding proteins with structural and/or functional relationship with lamin A/C (secondary laminopathies). Since 1994, when the first laminopathy, type 1 Emery-Dreifuss Muscular Dystrophy, was associated to the *EMD* gene [1] and much more since 1999, when *LMNA* mutations were linked to the type 2 form of the same muscular dystrophy [2], the interest in lamin and nuclear envelope role in disease has grown exponentially. Even more because, unexpectedly, more than ten different clinical entities including disorders of adipose tissue, cardiomyopathies, and accelerated ageing syndromes have been linked to *LMNA* mutations in the following years and at least the same number of diseases have been associated with mutations in other nuclear envelope genes. This has in turn pushed forward basic research in the field of lamin and nuclear envelope function, and international highly engaged and motivated teams around the world have provided exciting advances in the understanding of pathologies and their molecular basis.

The International Meeting on Laminopathies held in Bologna, Italy, on April 2017, brought together leading researchers in the field and initiated stimulated discussions and new cues for future research that we are presenting in the articles of this special issue of *Nucleus* on Laminopathies. We are extremely grateful to *Nucleus* and the colleagues who contributed the articles, and to all leading scientists and young researchers, who participated in the International Meeting on Laminopathies and shared their knowledge and ideas. We are also grateful to patients and their associations, who contributed their experience at the meeting and added knowledge to our understanding of diseases.

This issue has a clear interdisciplinary structure, as had the International Meeting on Laminopathies, not only because it includes papers with a prevailing clinical approach and others mostly referring to molecular and biological advances in the study of lamin-linked mechanisms, but also because all clinical phenotypes are taken into account and common symptoms (such as cardiomyopathy or lipodystrophy), are discussed across different laminopathies. Moreover, the outcome of a variety of different experimental approaches, from epigenetics to cell biology is reported in the articles addressing lamin-dependent pathogenetic pathways.

Clinical studies reported in the *Nucleus* issue provide an updated review of current knowledge of disease symptoms and management and are also intended as a starting point to collect more data and design new ad hoc studies to identify clinically useful predictors to stratify risk in mutation carriers, including probands and their asymptomatic relatives. Heart dysfunction, a symptom shared by Emery-Dreifuss Muscular Dystrophy, other neuromuscular laminopathies, Dilated Cardiomyopathy with conduction disease, as well as Hutchinson-Gilford Progeria and cases of Familial Partial Lipodystrophy, is the topic of several articles presenting new tools for early diagnosis, pharmacological and instrumental treatments and known pathomechanisms (Boriani, Brugada, Peretto). Aspects related to the age at onset and treatment of early onset forms of cardiolaminopathies are also discussed (Brugada). The Emery-Dreifuss muscular dystrophy neuromuscular phenotype is extensively described in the clinical papers, always with attention to the pathogenic mechanisms (Madey-Pilarzyck). An unexpected common feature of Emery-Dreifuss patients, the increase of circulating TGFbeta 2, and its effect on muscle and tendon cells are reported in the research article made possible by a wide collaborative study of the Italian Network for Laminopathies with the contribution of French researchers of the Muchir and Bonne group (Bernasconi). Papers on type 2 Familial partial lipodystrophy (Araujo-Vilar, Gambineri, Vigouroux) describe the most recent diagnostic criteria and therapeutic approaches and report interdisciplinary studies derived in long-lasting collaborations between clinicians and basic researchers that allowed the discovery of major players in disease mechanisms: defective nuclear lamina/nuclear envelope-dependent gene expression, prelamin A accumulation or altered chromatin conformation as the molecular basis of lipodystrophy symptoms common to Familial Partial Lipodystrophy and progeroid laminopathies. In this context, the paper of the Schirmer group as well as the Collas’ group article start to explain the tissue-specific pathogenic effect of some *LMNA* mutations. A tissue-specific pathogenic effect is also hypothesized for Emery-Dreifuss muscular dystrophy, based on epigenetic studies (Lanzuolo).

In any field of laminopathy research, preclinical models, from IPSCs to model organisms, provided relevant insights to the understanding of molecular and biological effects of lamin mutations, as also reported in this issue (Gruenbaum). Preclinical studies here reported show that complex chromatin rearrangements, which stem from either altered lamin interaction with epigenetic enzymes (Lanzuolo) or impaired nuclear lamina contacts with chromatin domains, may account for altered gene expression eliciting tissue-specific defects (Gruenbaum, Schirmer, Collas). Further, DNA replication defects, in terms of deprotection of replication forks, are identified in the paper from the Gonzalo group as contributing to the DNA damage that accumulates upon lamin dysfunction, while the relevance of protein platforms including lamins and newly identified functional partners is highlighted in the paper by Saggio. Even the most unexpected pathogenic events such as altered extracellular matrix deposition with fibrosis (Bernasconi, Vigouroux) or induction of inflammatory response have been identified in preclinical models and confirmed in clinical testing (Maggi).

Finally, most of the papers in this issue provide also an update on therapeutic perspectives for laminopathic patients and in particular the comprehensive paper by the Levy’s group summarizes and critically reviews treatment strategies for Hutchinson-Gilford Progeria, one of the most severe laminopathies and perhaps the best investigated for potential therapeutic strategies.

Laminopathy research is now moving towards this interdisciplinary approach, possibly the only promising strategy to find efficient treatment and a cure for these diseases. We believe that this *Nucleus* issue can represent a milestone to make clear where we are and provide new paths.
